# Genome-wide identification, evolution and expression pattern analysis of the *GATA* gene family in *Sorghum bicolor*

**DOI:** 10.3389/fpls.2023.1163357

**Published:** 2023-08-04

**Authors:** Xin Yao, Dili Lai, Meiliang Zhou, Jingjun Ruan, Chao Ma, Weijiao Wu, Wenfeng Weng, Yu Fan, Jianping Cheng

**Affiliations:** ^1^ College of Agronomy, Guizhou University, Guiyang, China; ^2^ Institute of Crop Science, Chinese Academy of Agriculture Science, Beijing, China

**Keywords:** *Sorghum bicolor*, *GATA* gene family, genome-wide identification, evolution, gene expression

## Abstract

The GATA family of transcription factors is zinc finger DNA binding proteins involved in a variety of biological processes, including plant growth and development and response to biotic/abiotic stresses, and thus play an essential role in plant response to environmental changes. However, the *GATA* gene family of *Sorghum* (*SbGATA*) has not been systematically analyzed and reported yet. Herein, we used a variety of bioinformatics methods and quantitative Real-Time Polymerase Chain Reaction (qRT-PCR) to explore the evolution and function of the 33 *SbGATA* genes identified. These *SbGATA* genes, distributed on 10 chromosomes, are classified into four subfamilies (I-IV) containing one pair of tandem duplications and nine pairs of segment duplications, which are more closely related to the monocot *Brachypodium distachyon* and *Oryza sativa GATA* genes. The physicochemical properties of the SbGATAs are significantly different among the subfamilies, while the protein structure and conserved protein motifs are highly conserved in the subfamilies. In addition, the transcription of SbGATAs is tissue-specific during *Sorghum* growth and development, which allows for functional diversity in response to stress and hormones. Collectively, our study lays a theoretical foundation for an in-depth analysis of the functions, mechanisms and evolutionary relationships of SbGATA during plant growth and development.

## 1 Introduction

Transcription factors (TFs) are a class of DNA-binding proteins which can not only bind to the promoter region of genes, but also form transcription factor complexes with other transcription factors to regulate the transcriptional activity of the target gene (Riechmann et al., 2000; Fan et al., 2021a; Lai et al., 2022a). Plant transcription factors participate in various physiological and biochemical pathways during the development of higher plants, such as plant growth and development (Strader et al., 2022), metabolic network (Rueda-López et al., 2015), stress response (Yao et al., 2022b), and disease resistance (Li et al., 2017). Plant transcription factor GATAs are a key class of factors that regulate various biological processes such as light response regulation, chlorophyll synthesis and environmental response in plants. The GATAs consist of one or two highly conserved type IVb zinc finger structural modules C-X_2_-CX_17-20_-C-X_2_-C (C, cysteine; X, other residues) (Schwechheimer et al., 2022), which binds to the 5’-WGATAR-3’ region (W, thymidine (T)/adenosine (A), R= guanidine (G)/adenosine SZAS (A)) of the target gene and activates or represses the transcription, thereby regulating plant growth and development (Guo et al., 2021). For example, after BRZ1 (BR-activated transcription factor 1) receptor sensing the BR (brassinosteroid) signals, the *GATA2* expression is repressed. Meanwhile, light affects plant seedling growth by inhibiting the hydrolysis of the photomorphogenesis-related factor COP1 (CONSTITUTIVE PHOTOMOR PHOGENESIS 1) to activate *GATA2* expression and feedback repression of GATA2 transcription, thereby affecting plant seedling growth (Luo et al., 2010). GNC (Nitrate-inducible, carbon metabolism-involved, GATA21), a member of the GATA family of transcription factors, is involved in chlorophyll synthesis and glucose regulation. Loss-of-function GNC mutants causes both reduced chlorophyll levels and altered glucose content (Rolland et al., 2002; Bi et al., 2005). Bhardwaj et al. (2015) identified five *GATAs* from mustard-type oilseed rape (*Brassica juncea*) and found that all of the five had decreased expression under drought stress, while two of them had downregulated and upregulated respectively under heat stress. AGP1 (AG-motif binding Protein), a GATA protein in tobacco (*Nicotiana tabacum*), binds to the NtMyb2 promoter region and regulates the expression of the phenylalanine ammonia lyase gene (PAL) in response to biotic stress (Sugimoto et al., 2003).

*Sorghum bicolor* (L.) Moench, a monocotyledonous plant of genus *Sorghum* in the *Gramineae* and *Andropogoneae* family, is a typical diploid crop (2n=20) widely grown worldwide and is the fifth most productive cereal crop after wheat, maize, rice and barley (Shrestha et al., 2021; Xu et al., 2021). As an annual C4 self-pollinated crop, sorghum has higher photosynthetic efficiency and higher biological yield than other energy crops (Li et al., 2013; Yang et al., 2020a). As a dry grain crop, *Sorghum* is widely adaptable, growth-competent, and tolerant of infertile conditions. Sorghum can be planted and grown under arid and semi-arid conditions and still maintain high yields and benefits, as well as survive cold, waterlogged conditions for short periods of time (Khoddami et al., 2023). *Sorghum* has become an important raw material for livestock feed, brewing, biofuels, industrial starch, and paper production (Ordonio et al., 2016; Ouyang et al., 2021; Fang et al., 2022).

Currently, the GATA gene family has been extensively studied in rice (Gupta et al., 2017), *Arabidopsis thaliana* (Reyes et al., 2004), *Fagopyrum tataricum* (Yao et al., 2022a) and *Brachypodium distachyon* (Peng et al., 2021) because of their critical growth and development function of plants. However, systematical analysis of the sorghum *GATA* gene family remains exclusive. Therefore, we identified the members of the sorghum *GAT*A gene family at the genome level by bioinformatics and other methods for the first time. We further analyzed their physicochemical properties, gene structure, chromosome distribution, cis-acting elements, and developmental evolutionary relationship within this gene family. More importantly, we studied the tissue specificity and fruit development of the *GATA* gene members of different subfamilies and found their expression patterns under different abiotic stresses and hormone treatments during seedling germination, which provides a better understanding of the evolutionary relationship among SbGATA transcription factors. In this study, the physical and chemical properties, evolutionary relationships and expression patterns of the *GATA* gene family in sorghum were systematically investigated by bioinformatics methods to provide a theoretical basis for an in-depth analysis of the biological functions, mechanisms of action and evolutionary relationships of the *GATA* gene family in sorghum.

## 2 Materials and methods

### 2.1 Identification of the GATA of *Sorghum bicolor*

The sorghum whole gene sequence and gene annotation files from the Phytozome website (https://phytozome-next.jgi.doe.gov/) and the Hidden Markov model (HMM) file for the GATA structural domains (PF00032) from the Pfam protein family database (Finn et al., 2011) were firstly downloaded and obtained. All possible SbGATA proteins from the sorghum genome were then de-redundantly screened by comparing to the GATA amino acid sequences downloaded from arabidopsis (https://www.arabidopsis.org/) and rice (http://rice.uga.edu/) using the BLASTp (score value ≥100, e-value ≤ 1e−10) (Altschul et al., 1997). Finally, CD-Search (https://www.ncbi.nlm.nih.gov/Structure/cdd/wrpsb.cgi) and SMART (http://smart.embl-heidelberg.de/smart/set_mode.cgi?NORMAL=1) were used to search for conserved structural domains in the sorghum genome, and sequences that did not contain conserved GATA domains were removed (Letunic and Bork, 2018; Yang et al., 2020b).

### 2.2 Analysis of the physicochemical properties of SbGATA proteins

The physicochemical properties of all identified SbGATA proteins were analyzed using the ExPASy website (https://www.expasy.org/), including molecular weight (MW), theoretical isoelectric point (pI) and instability index (II). Subcellular localization of the SbGATA proteins were also predicted by WoLF PSORT online website (https://wolfpsort.hgc.jp/) accordingly Yang et al. (2020c).

### 2.3 Gene structure, conserved motifs, cis-acting elements and protein−protein interactions

Multiple sequence alignment analysis of sorghum and arabidopsi*s* GATA families was performed using MEGA 11 software based on ClustalW default parameters (Thompson et al., 2002). The *SbGATA* gene structure map was constructed from sorghum genomic data using TBtools v1.0987663 software (Chen et al., 2020). The conserved motifs of the SbGATA proteins were predicted using the MEME online website (https://meme-suite.org/meme/tools/meme) with the maximum conserved motif search value set to 10 AA and the remaining parameters set to default values (Bailey et al., 2009). The PlantCARE online website (http://bioinformatics.psb.ugent.be/webtools/plantcare/html/) was used to predict the cis-acting elements in the *SbGATA* promoter sequence (upstream 2000 bp). The protein interaction networks of SbGATAs were established through the STRING protein interaction database (https://cn.string-db.org/).

### 2.4 Chromosomal location, duplication events and syntenic analysis

Referring to the method of Krzywinski et al. (2009), the *SbGATA* gene was localized to ten chromosomes of sorghum based on gene localization information from the sorghum genome file. Tandem duplications and segment duplication of *SbGATA* genes were analyzed with Multiple Collinearity Scan Toolkit X (MCScanX) and default parameters (Wang et al., 2012). Homology between *Sorghum bicolor* and other six species (*Arabidopsis thaliana*, *Solanum lycopersicum*, *Vitis vinifera*, *Glycine max*, *Brachypodium distachyon* and *Oryza sativa*) was analyzed with Dual Synteny Plotter (Chen et al., 2020).

### 2.5 Phylogenetic evolution and classification of SbGATA family

The GATA amino acid sequences (**Table S7**) of six species (*O. sativa*, *B. distachyon*, *G. max*, *V. vinifera*, *S. lycopersicum*, and *A. thaliana*) (Zhang et al., 2015; Gupta et al., 2017; Yuan et al., 2018; Zhang et al., 2018; Wang et al., 2019; Peng et al., 2021) were obtained from references on GATA that has identified different crops. The Muscle Wrapper model was used to align the GATA amino acid sequences of seven species (*S. bicolor*, *O. sativa*, *B. distachyon*, *G. max*, *V. vinifera*, *S. lycopersicum*, and *A. thaliana*). The phylogenetic tree was further constructed by the IQ-Tree Wrapper program in TBtools v1.0987663 software. The boostrap number was set to 1000, and other parameters were default. The evolutionary tree between *S. bicolor* and *A. thaliana* was constructed as above, and the identified SbGATAs were classified and grouped according to the model plant *A. thaliana*.

### 2.6 Plant materials, growth and treatments in *Sorghum bicolor*

The sorghum variety ‘Hong Ying Zi’, which was preserved by the group, were used for the following experiments. The seeds were grown in a greenhouse under growth conditions of 16 h/25°C, 8 h/16°C and 75% relative humidity. Six abiotic stresses (Cold, 4°C; Dark, complete shading; Flooding, whole plant; Heat, 40°C; NaCl, 150 mmol·L^−1^; PEG: 30%) and four hormone treatments (ABA, 100 μmol·L^−1^; GA,100 μmol·L^−1^; MeJA, 100 μmol·L^−1^; SA, 100 μmol·L^−1^) were applied to uniformly grown sorghum seedlings when they reached the three-leafed stage. Each treatment was triplicated and corresponding sorghum seedlings were sampled at 0 h, 3 h, 12 h and 24 h. Samples were collected from the root, stem, young leaf, mature leaf, flower, and from the fruit and husk at the early, middle and late of grain-filling stage, and then stored at -80°C before further usage.

### 2.7 Total RNA extraction and cDNA synthesis

Total RNA was extracted from sorghum samples using the E.Z.N.A. Plant RNA Kit (Omega Bio-Tek, Inc, USA). The RNA integrity was examined by electrophoresis on a 1% agarose gel, and RNA concentration and quality were determined using an ultra-micro spectrophotometer (Beijing Kaiao Technology Development Co, Ltd., China). The cDNA was synthesized according to the instructions of HiScript II Q RT SuperMix for qPCR Kit (Vazyme Biotech Co., Ltd, China).

### 2.8 Quantitative real-time polymerase chain reaction analysis

Primer Premier 5.0 software (Premier, Canada) was used to design qRT-PCR specific primers for the 8 *SbGATA* genes (**Table S8**) with product lengths of 80-200 bp, and *SbUBQ10* (*actin* gene of *S. bicolor*) was used as an internal reference gene. The qRT-PCR method was based on the ChamQ Universal SYBR qPCR Master Mix Kit (Vazyme Biotech Co., Ltd, China). Amplification was performed using the CFX96 Real-Time System instrument (BIO-RAD, USA). The ChamQ Universal SYBR qPCR master mix kit (Vazyme Biotech Co., Ltd, Nanjing, China) was used with 1.0 μL cDNA, 10.0 μL 2×SYBR mix, 0.4 μL of each primer, and 8.2 μL ddH_2_O. The reaction process was 40 cycles at 95°C for 3 min, 95°C for 5 s, and 55°C for 30 s, and one cycle at 95°C for 10 s, 60°C for 60 s, and 95°C for 15 s. The relative expression of genes was calculated using the 2^-ΔΔCt^ formula (Livak and Schmittgen, 2001). Three biological replicates and three technical replicates were set up.

## 3 Results

### 3.1 Identification of SbGATA family

Using the GATA amino acid sequences of *A. thaliana* and *O. sativa* as references, we searched for the SbGATA in the *S. bicolor* genome database by BLASTp alignment. After HMMER, CD-Search and SMART analysis, a total of 33 *SbGATA* genes were identified and named *SbGATA01*-*SbGATA33* based on their physical position on chromosomes (**Table S1**; **Table 1**). The molecular weight (MW), theoretical isoelectric point (pI), instability index (II) and subcellular localization of these 33 SbGATAs were analyzed (**Table 1**). The coding sequence (CDS) length of the 33 *SbGAT*A genes ranged from 378 to 2184 bp, and the corresponding amino acid length of their proteins ranged from 125 to 727 AA, of which SbGATA12 had the shortest sequence and SbGATA08 had the longest (**Table 1**). The molecular weight (MW) of the 33 SbGATA proteins ranged from 13.61 to 82.18 kilodalton (kD). Obviously, SbGATA12 had the smallest MW while SbGATA08 had the largest (**Table 1**), which was consistent with their length of CDS and amino acid sequences, indicating that MW is positively proportional to gene sequence length and vice versa. Surprisingly, the isoelectric point (pI) of the 33 SbGATA proteins dramatically distributed between 4.60 (SbGATA04) and 11.63 (SbGATA09), with most of them (24/33) having a pI greater than 7 and mainly concentrated between 7~9 (22/24) (**Table 1**), suggesting that the SbGATA family proteins tend to be enriched in basic amino acids. We also analyzed the instability index (II) of the 33 SbGATA proteins and found that the instability index of all 33 SbGATAs was greater than 40 (**Table 1**), with SbGATA09 having the largest index (81.28), indicating that they need other regulators to form stable complex to perform their function. As expected, the predicted subcellular localization of most SbGATA proteins (26/33) were in nuclear, while five SbGATAs (SbGATA18, SbGATA22, SbGATA23, SbGATA30 and SbGATA31) were in the chloroplast, and two SbGATA (SbGATA05 and SbGATA13) were in the mitochondria (**Table 1**).

**Table 1:** List of the *SbGATA* genes identified in the study.

Gene name	Accession number/Gene ID	Chromosome	Coding sequence (CDS)/bp	Encoded protein					
				Amino acid length/aa	Molecular weight (MW)/kD	Theoretical isoelectric point (pI)	Instability index (II)	Subcellular localization	Subfamily
*SbGATA01*	SORBI_3001G023900	Chr 1	471	156	17.02	9.98	78.86	nuclear	II
*SbGATA02*	SORBI_3001G062700	Chr 1	1104	367	38.07	6.99	58.07	nuclear	I
*SbGATA03*	SORBI_3001G100100	Chr 1	831	276	29.63	8.95	54.69	nuclear	III
*SbGATA04*	SORBI_3001G135400	Chr 1	912	303	32.44	4.60	62.41	nuclear	III
*SbGATA05*	SORBI_3001G137300	Chr 1	1341	446	48.69	6.55	60.46	mitochondria	IV
*SbGATA06*	SORBI_3001G229200	Chr 1	1374	457	47.99	9.90	57.15	nuclear	IV
*SbGATA07*	SORBI_3001G299600	Chr 1	1155	384	39.35	7.78	51.31	nuclear	I
*SbGATA08*	SORBI_3001G482200	Chr 1	2184	727	82.18	7.12	43.82	nuclear	IV
*SbGATA09*	SORBI_3001G506066	Chr 1	555	184	19.38	11.63	81.28	nuclear	I
*SbGATA10*	SORBI_3002G018250	Chr 2	714	237	27.10	9.20	49.99	nuclear	IV
*SbGATA11*	SORBI_3002G374300	Chr 2	2112	703	80.11	7.30	48.76	nuclear	IV
*SbGATA12*	SORBI_3003G157300	Chr 3	378	125	13.61	9.80	69.58	nuclear	II
*SbGATA13*	SORBI_3003G246800	Chr 3	735	244	24.61	7.45	51.79	mitochondria	II
*SbGATA14*	SORBI_3003G293100	Chr 3	1239	412	42.45	5.51	59.08	nuclear	I
*SbGATA15*	SORBI_3003G445800	Chr 3	654	217	23.95	9.55	61.94	nuclear	II
*SbGATA16*	SORBI_3004G023500	Chr 4	870	289	30.66	6.65	75.21	nuclear	I
*SbGATA17*	SORBI_3004G094100	Chr 4	1164	387	41.66	8.78	75.48	nuclear	II
*SbGATA18*	SORBI_3004G301100	Chr 4	1434	477	49.75	8.40	63.25	chloroplast	I
*SbGATA19*	SORBI_3004G337500	Chr 4	1305	434	44.72	5.66	68.19	nuclear	I
*SbGATA20*	SORBI_3005G022400	Chr 5	1425	474	48.38	8.83	79.16	nuclear	I
*SbGATA21*	SORBI_3005G162400	Chr 5	393	130	14.69	9.73	53.15	nuclear	III
*SbGATA22*	SORBI_3006G162800	Chr 6	1356	451	47.44	8.05	69.83	chloroplast	I
*SbGATA23*	SORBI_3006G166100	Chr 6	1629	542	59.30	6.35	54.04	chloroplast	IV
*SbGATA24*	SORBI_3008G051400	Chr 8	1326	441	47.34	10.12	73.34	nuclear	I
*SbGATA25*	SORBI_3008G051500	Chr 8	1191	397	42.75	9.32	80.91	nuclear	I
*SbGATA26*	SORBI_3008G051700	Chr 8	1644	547	58.61	9.00	61.87	nuclear	I
*SbGATA27*	SORBI_3008G179800	Chr 8	909	302	33.84	8.93	61.77	nuclear	I
*SbGATA28*	SORBI_3009G050600	Chr 9	471	156	17.38	9.76	72.50	nuclear	II
*SbGATA29*	SORBI_3009G202000	Chr 9	1239	412	42.18	5.81	59.44	nuclear	I
*SbGATA30*	SORBI_3009G236000	Chr 9	690	229	24.11	7.43	78.55	chloroplast	II
*SbGATA31*	SORBI_3009G243600	Chr 9	891	296	30.32	7.85	52.87	chloroplast	II
*SbGATA32*	SORBI_3010G173400	Chr 10	1161	386	40.72	8.50	61.38	nuclear	II
*SbGATA33*	SORBI_3010G249200	Chr 10	1059	352	37.51	5.12	50.54	nuclear	III

### 3.2 Phylogenetic analysis, classification and multiple sequence alignment of SbGATAs proteins

**Figure 1:**
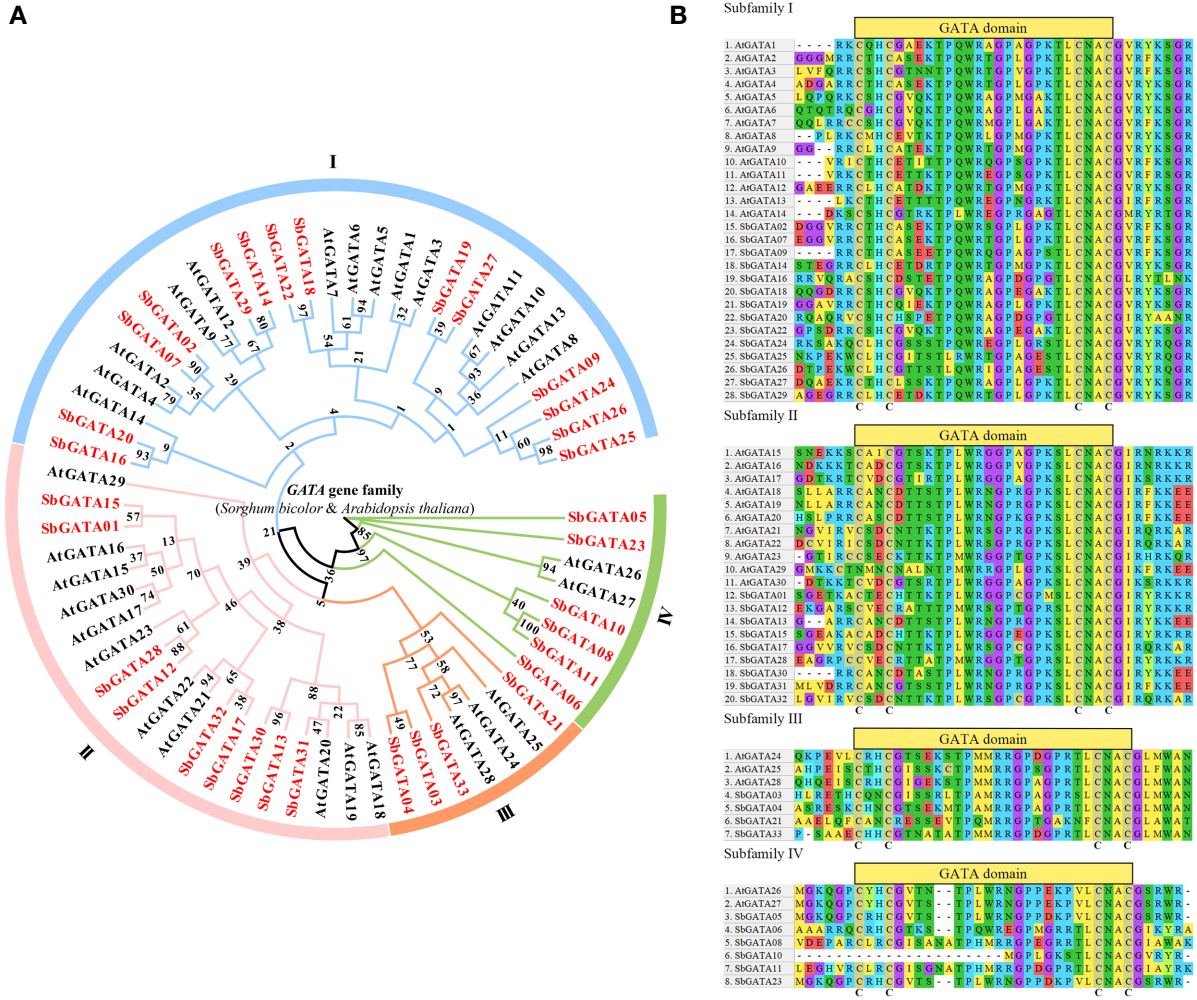
**(A)** Phylogenetic tree of the GATA proteins of *S. bicolor* and *A. thaliana* family. I, II, III and IV represent different subfamilies. **(B)** Multiple sequence alignment of the GATA structural domains of four subfamilies of *S. bicolor* and *A. thaliana* using 40 AA. The C (cysteine) represented the highly conserved type IVb zinc finger structure.

Using the 33 identified SbGATA proteins and other 30 reported *A. thaliana* GATA (AtGATA) proteins, we constructed a phylogenetic tree. The 33 SbGATAs were classified into four subfamilies (I, II, III and IV) according to the classification of AtGATAs (**Figure 1A**). Among them, subfamily I contained the most SbGATA members (14/33), followed by subfamily II (9/33), subfamily IV (6/33), and then subfamily III (4/33) (**Figure 1A**). It is worth noting that SbGATA05 and SbGATA23 form a separate branch in subfamily IV (**Figure 1A**), indicating that they are in an evolutionary transition state in subfamily IV.

To further investigate the evolutionary relationship between the structural domains of SbGATA proteins in different subfamilies, the amino acid sequences of all SbGATAs and AtGATAs were analyzed by multiple sequence alignment, and 40 amino acid sequences containing the GATA domain were selected for analysis (**Figure 1B**). The majority of SbGATA proteins contain a single GATA domain, while a few SbGATA proteins in subfamilies I (SbGATA24, 25, 26 and 27) and IV (SbGATA06) had double GATA domains (**Figure 1B**). Moreover, the conserved domains of subfamilies I and II conform to the zinc finger structure of CX_2_CX_18_CX_2_C, in which SbGATA25 and SbGATA26 had the zinc finger structure of CX_2_CX_19_CX_2_C (**Figure 1B**). The subfamily III contained the CX_2_CX_20_CX_2_C zinc finger structure (**Figure 1B**). In subfamily IV, SbGATA08 and SbGATA11 had the CX_2_CX_20_CX_2_C structure, while SbGATA10 lacked the CX_2_C structure, which may have a new function distinct from the other subfamily IV members (**Figure 1B**). The remaining three SbGATA proteins in subfamily IV all contained a typical CX_2_CX_18_CX_2_C structure (**Figure 1B**). In addition, we found that all the four subfamilies contain some highly conserved motifs such as GP and CNAC (**Figure 1B**), although there were some other conserved motifs between different subfamilies and some differences in the GATA domain among the same subfamily. Among them, the 30th amino acid of SbGATA21 in subfamily III was Phenylalanine (Phe, F), which was distinct from the Leucine (Leu, L) of all other GATA proteins (**Figure 1B**). In subfamily IV, the GATA domains of SbGATA05 and SbGATA23, which were individually branched in the phylogenetic tree, were identical, and SbGATA08 and SbGATA11 have an additional NA sequence (9-10 amino acids) (**Figure 1B**).

### 3.3 Structures and conserved motifs analysis of SbGATA family

**Figure 2:**
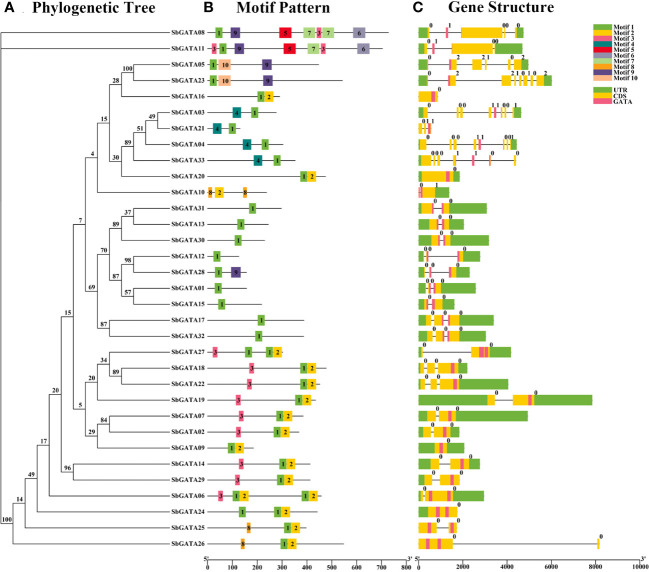
Phylogenetic tree, motif pattern and gene structure of GATA genes in *Sorghum bicolor*. **(A)** The phylogenetic tree is constructed by the full-length sequences of *Sorghum bicolor* GATA proteins with 1000 replicates on each node. **(B)** The amino acid motifs (numbered 1-10) in SbGATAs are displayed in ten colored boxes, and black lines indicate protein sequence length. **(C)** Green rectangles, yellow rectangles, pink rectangles and black lines represent the UTR (untranslated region), CDS (coding sequence or exons), *GATA* domain and introns, respectively.

To analyze the diversity of sorghum GATAs during evolution, the conserved motifs of 33 SbGATA proteins were analyzed using the MEME online website, and a composite map of the phylogenetic tree, motif patterns, and protein structures of the 33 SbGATAs was constructed from sorghum genome annotation files using TBtools (**Figures 2A–C**; **Table S2**). **Figure 2B** showed that, with the exception of a few sorghum GATAs, GATA proteins of the same subfamily or subgroup contain similar motifs. For example, all proteins except SbGATA10 contain motif 1, namely GATA motif; in subfamily I, most proteins have three motifs (10/14); in subfamily II, the number of motifs is relatively small, with most proteins having only one motif (8/9); in subfamily III, all four SbGATAs had two motifs, and motif 1 was positioned after motif 4 in the amino acid sequence (**Figure 2B**). The differences in the number and variety of conserved motifs in *Sorghum* GATA proteins reflect the structural diversity of these proteins, while predicting that they have different biological functions.

**Figure 3:**
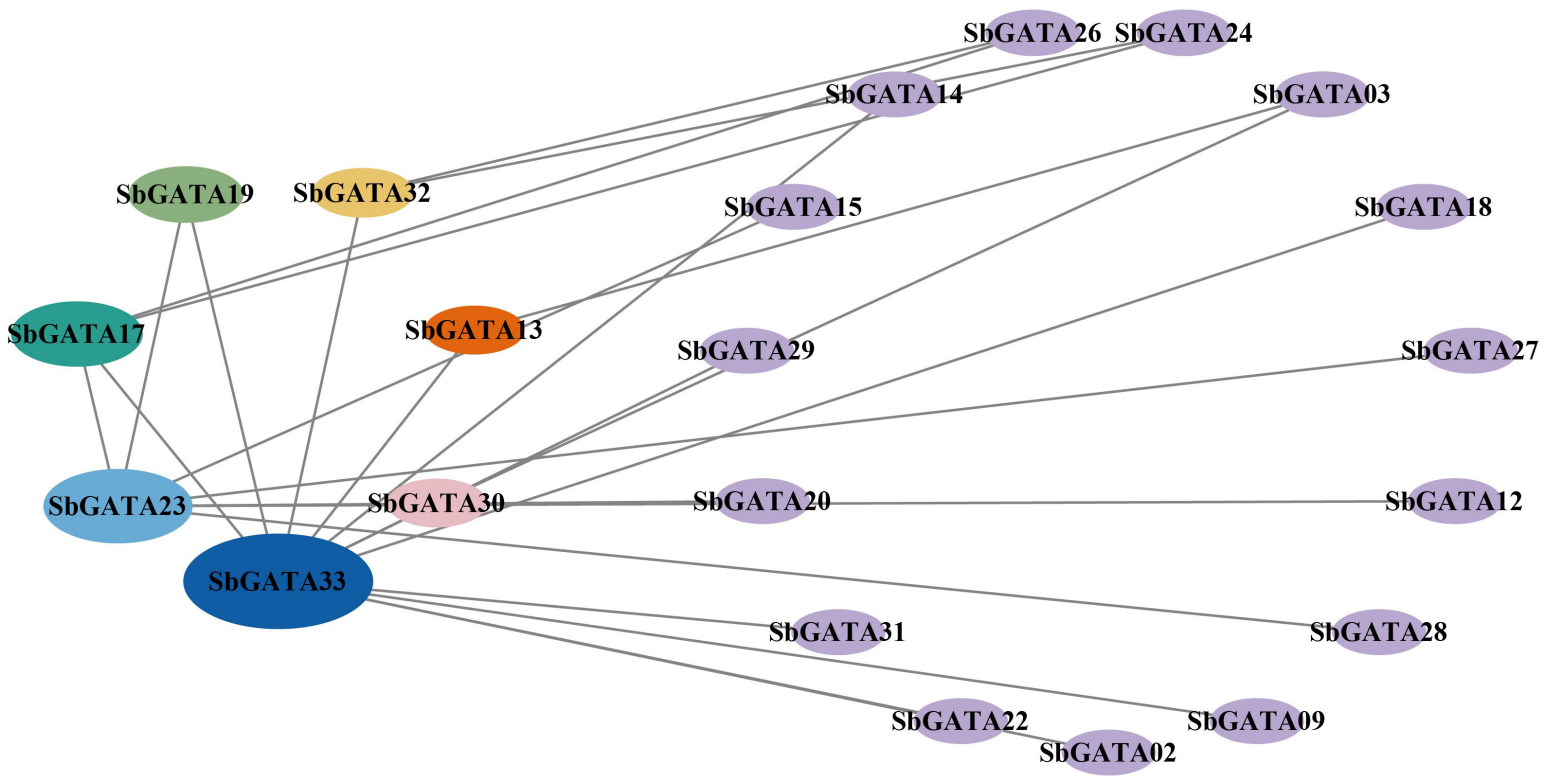
Predicted results of the protein−protein interaction network among 33 SbGATA proteins. The lines indicate the interactions between two SbGATA proteins. The ellipses indicate the SbGATA proteins, and the larger the ellipse shape, the more proteins interact with it. The different colors of the ellipses represent different degrees of interaction.

Therefore, the interactions between the 33 identified SbGATA proteins were predicted through the STRING online website (**Figure 3**). We identified 22 SbGATA proteins that may interact with each other. Of these, 11 and 8 SbGATAs belong to subfamilies I and II, respectively, while subfamily III (SbGATA03 and SbGATA33) and subfamily IV (SbGATA23) have fewer proteins interacting with them (**Figure 3**).

Based on the sorghum genome sequence, a gene structure map of sorghum *GATAs* was constructed (**Figure 2C**). The results showed that *SbGATA10* contains only one CDS region, while the remaining 32 *SbGATAs* all contain no less than 2 CDS regions (**Figure 2C**). Among them, subfamilies I and II both contain 2-4 CDSs, while *SbGATA23* in subfamily IV have the 8 CDS segments, which is the most abundant (**Figure 2C**). For the untranslated region (UTR), *SbGATA*16, 21, 25 and 26 have no UTRs, and the remaining *GATA*s have their UTRs distributed almost at both ends of the gene (**Figure 2C**). In addition, *SbGATA*09, 16, 20 and 24 have no intron, *SbGATA*26 has the longest intron, and all the five genes belong to subfamily I (**Figure 2C**), which indirectly indicated that the more CDS segments in the gene sequence, the more introns.

### 3.4 Cis-regulatory elements analysis and protein−protein interactions of SbGATA family

**Figure 4:**
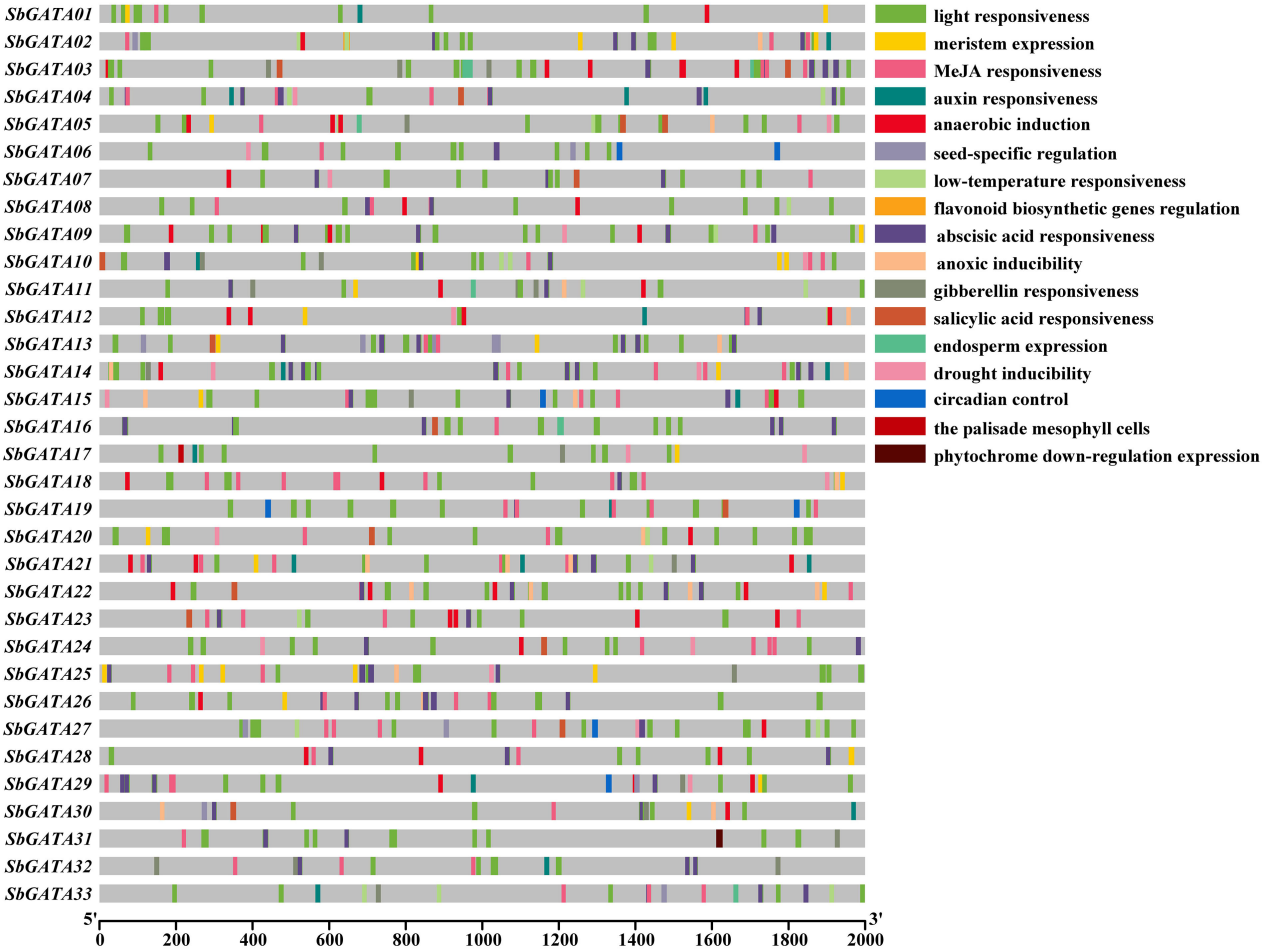
Cis-acting elements of the promoter region (upstream 2000 bp) of 33 *GATA* genes in *Sorghum bicolor*.

In this paper, 2000 bp upstream of each *SbGATA* was selected as the promoter sequence and corresponding cis-acting elements, mainly including hormone response elements, abiotic stress response elements, and physiological and biochemical metabolism response elements, were predicted through the PlantCARE online website (**Figure 4**; **Table S3**). The results showed that among the 33 *SbGATA* promoter sequences, the light-responsive element with a count of 377 is the most abundant and widely distributed in all promoter sequences, followed by abscisic acid responsive element with a count of 123, which is distributed in the vast majority of the promoter sequences (**Figure 4**). We also found that the promoter sequence of this family contains five hormone-responsive elements, such as abscisic acid-responsive, MeJA-responsive, auxin-responsive, gibberellin-responsive and salicylic acid-responsive elements, and contains two abiotic response elements, such as low-temperature-responsive and drought-inducible elements (**Figure 4**). Among the hormone-responsive elements, abscisic acid responsiveness (123 counts) and MeJA responsiveness (87 counts) were more numerous, while the abiotic stress-responsive elements, namely low-temperature responsiveness (21 counts) and drought inducibility (19 counts), were much less numerous (**Figure 4**). Among the 33 sorghum GATAs promoter sequences, *SbGATA10* distributed five hormone response elements and two abiotic response elements (**Figure 4**), suggesting that this gene may have multiple physiological and biochemical regulatory mechanisms under stress conditions.

### 3.5 Chromosomal location, duplication events and synteny analysis of *SbGATAs*

**Figure 5:**
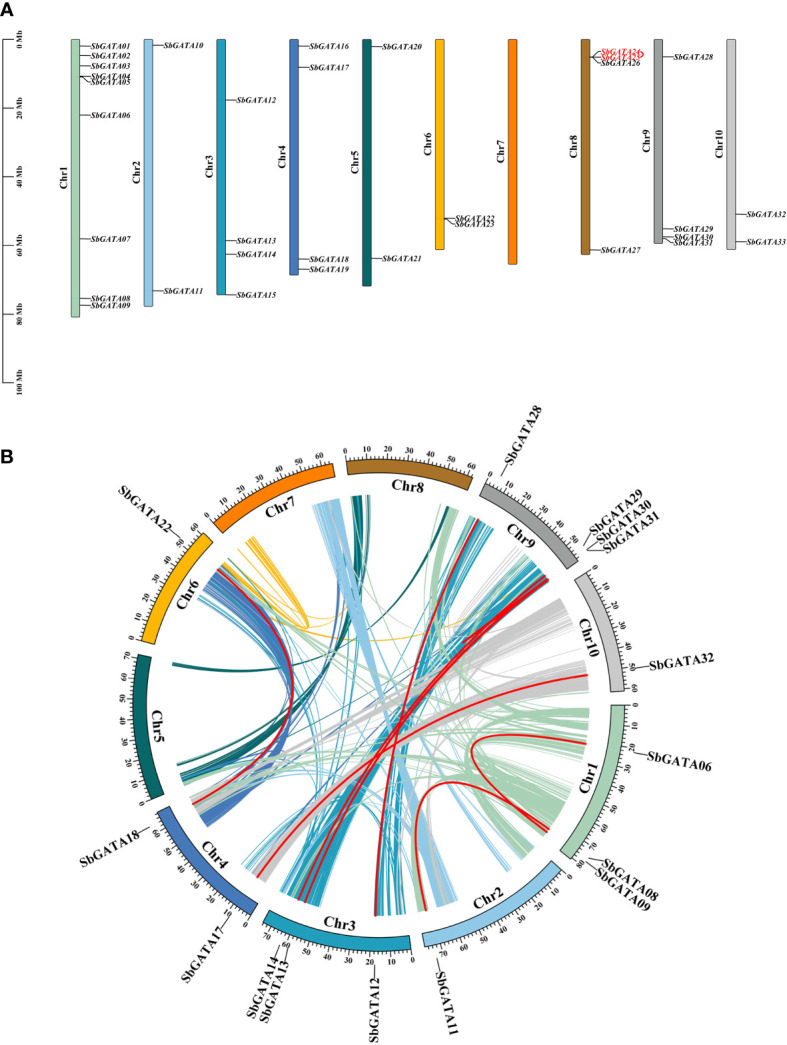
**(A)** Chromosomal location of *GATA* genes in *Sorghum bicolor*. The colored rectangular bars represent the chromosomes of *Sorghum bicolor*, and the 0–100 Mb scale represents chromosome length; the Chr 1-10 represents each corresponding chromosome; red fonts represent gene tandem duplications. **(B)** Collinearity of *GATA* genes in *Sorghum bicolor*. Colored lines indicate the all syntenic blocks in the genomes of *Sorghum bicolor*, and red lines indicate duplicated *GAT*A gene pairs. The chromosome number is shown inside each chromosome.

Sorghum *GATA* genes are unevenly distributed on 10 chromosomes, and *GATA* genes of the same subfamily are also randomly distributed on the chromosomes (**Figure 5A**). Of these, chromosome 1 (Chr 1) contains the most *SbGATAs* (9/33, 27.27%), followed by Chr 3, Chr 4, Chr 8 and Chr 9 all with four *SbGATAs* genes (4/33, 12.12%), Chr 2, Chr 5, Chr 6 and Chr 10 all distributed with two *SbGATA* genes (2/33, 6.16%), while Chr 7 has no distribution of *SbGATA* genes (**Figure 5A**). In addition, the four *SbGATAs* distributed in Chr 9, which all belong to subfamily I. Only one pair of the 33 *SbGATA* genes (*SbGATA24* and *SbGATA25*) is found to be tandemly duplicated on Chr 8 (**Table S4**), and these two genes all belong to subfamily I (**Figure 5A**).

In this study, we analyzed gene duplication events of the 33 *SbGATA* genes (**Figure 5B**) and identified 16 homologous loci and 9 pairs of quasi-homologous *GATAs* in the sorghum genome originated from large segmental duplication, namely *SbGATA06*/*SbGATA09*, *SbGATA08*/*SbGATA11*, *SbGATA17*/*SbGATA32*, *SbGATA13*/*SbGATA31*, *SbGATA13*/*SbGATA30*, *SbGATA14*/*SbGATA29*, *SbGATA12*/*SbGATA28*, *SbGATA18*/*SbGATA22* and *SbGATA30*/*SbGATA31*, of which five pairs belonged to subfamily II. *SbGATAs* were unevenly distributed among the 10 linked regions (LG) of the sorghum genome, with LG9 containing the largest number of *SbGATAs* (4/16), followed by LG3 containing three *SbGATAs* (3/16), while LG5, LG7 and LG8 had no duplicated genes (**Figure 5B**).

**Figure 6:**
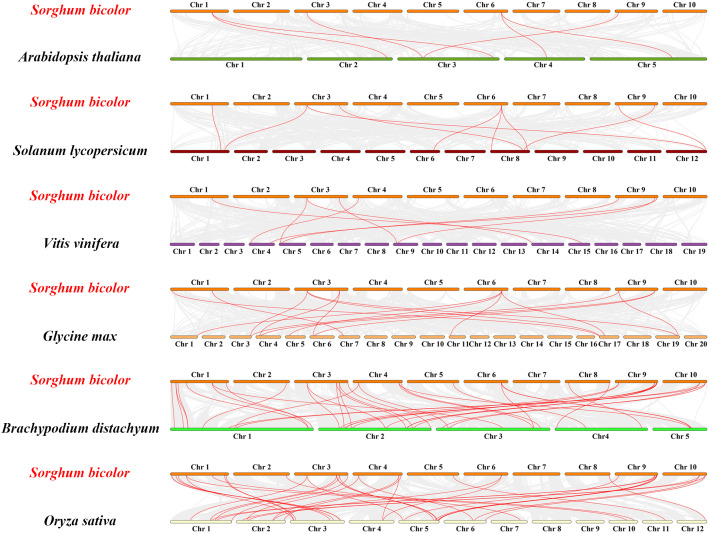
Synteny analysis of GATA genes between Sorghum bicolor and six other plants (*Arabidopsis thaliana, Solanum lycopersicum, Vitis vinifera, Glycine max, Brachypodium distachyon* and *Oryza sativa*). The gray lines between *Sorghum bicolor* and the other plant represent synteny blocks in the wide genomes of *Sorghum bicolor* and other plants, while red lines indicate the orthologous relationship of GATA genes.

The occurrence of gene duplication and segmental duplication events can expand the number of gene family members, which is an important driver of gene family expansion, as well as a major driving force of species evolution. To explore the evolutionary relationships between SbGATAs and different species, we constructed interspecific synteny map of *S. bicolor* with other six representative plants, including three monocotyledons (*S. lycopersicum*, *B. distachyon* and *O. sativa*) and three dicotyledons (*A.thaliana*, *V vinifera* and *G max*) (**Figure 6**; **Table S5**). From the synteny map, we noticed that the SbGATA genes had higher synteny with the GATA genes of monocotyledons since *S.bicolor* has 39 pairs of synteny genes, which was the highest number, with either *B distachyon* or O *sativa* (**Figure 6**). In contrast, the *S. bicolor* GATA gene family had fewer gene pairs in common with dicotyledons, with only eight and six pairs of genes in common with *V vinifera* and *A thaliana*, respectively (**Figure 6**). We also found that *SbGATA07*, *SbGATA12* and *SbGATA28* had synteny homologs with all six plants, *SbGATA14* and *SbGATA29* have synteny genes with all three monocotyledons, whereas *SbGATA02*, *SbGATA05*, *SbGATA08*, *SbGATA10*, *SbGATA16*, *SbGATA20*, *SbGATA25* and *SbGATA26* had no synteny genes with any of the six plants (**Figure 6**).

### 3.6 Evolutionary analysis of *Sorghum bicolor* GATAs with other plants

**Figure 7:**
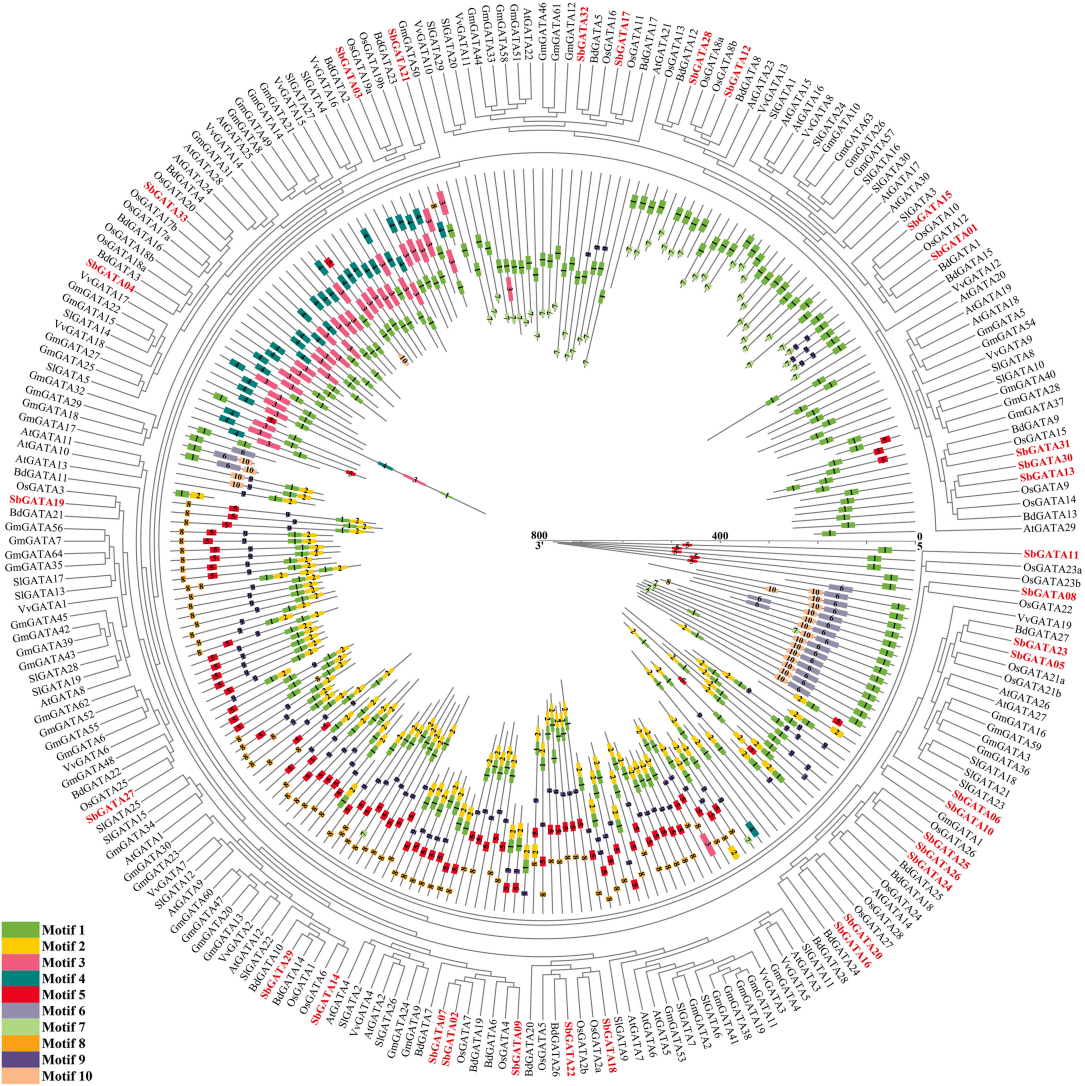
Phylogenetic relationships and motif pattern of GATAs protein among *Sorghum bicolor* and six other plants (*Arabidopsis thaliana*, *Solanum lycopersicum*, *Vitis vinifera*, *Glycine max*, *Brachypodium distachyon* and *Oryza sativa*). The colored legends represent amino acids motifs (numbered 1-10), the outer part of the circle represents the phylogenetic tree of GATA proteins from the seven plants, and the inner part of the circle represents protein lengths, conserved motifs and their composition. The red fonts represent the 33 SbGATAs.

In order to further study the genetic relationship between *S. bicolor* GATAs and GATAs from other plants, an interspecific evolutionary tree was constructed by comparing the amino acid sequences of 33 SbGATA proteins and GATAs from the abovementioned six plants (*A. thaliana*, *S. lycopersicum*, *V. vinifera*, *G. max*, *B. distachyon* and *O. sativa*) (**Figure 7**; **Tables S6**, **S7**). Obviously, SbGATAs were closely clustered with *B. distachyon* and *O. sativa* GATAs (**Figure 7**), indicating that SbGATAs were more closely related to monocotyledonous GATAs. All GATAs were distributed with motif 1, suggesting that motif 1 was a conserved motif of GATA. SbGATA06, SbGATA24, SbGATA25, SbGATA26 and SbGATA27 were even have two motif 1 (**Figure 7**). Meanwhile, SbGATAs in the same subfamily had similar motifs, while motifs in different subfamilies vary considerably. For example, subfamily I had the motifs 5-9-1-2 while subfamily III had the motifs 3-1 (**Figure 7**). There were also motifs that had different distributions within the same subfamily. For example, SbGATA13, SbGATA30 and SbGATA31 in subfamily II only contain motif 1, and IV there was more variation among SbGATAs within subfamily IV (**Figure 7**).

### 3.7 Transcriptional activity of *SbGATA* genes in different tissues

**Figure 8:**
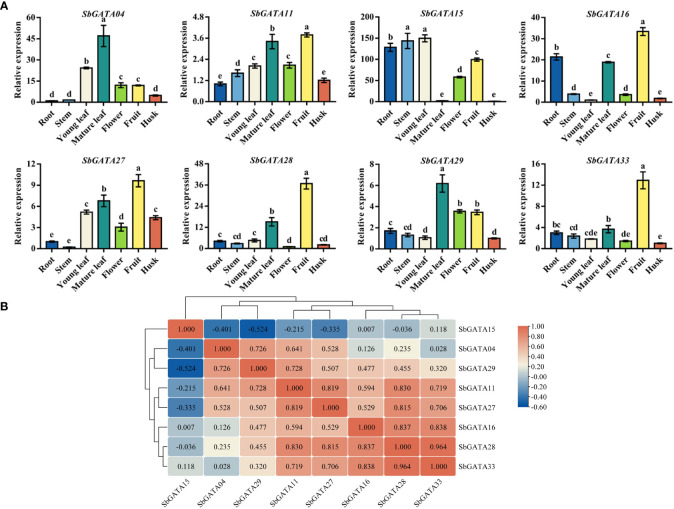
Relative transcriptional activity and corresponding correlation analysis of eight *SbGATAs* including *SbGATA04*, *SbGATA11*, *SbGATA15*, *SbGATA16*, *SbGATA27*, *SbGATA28*, *SbGATA29* and *SbGATA33*. **(A)** Relative transcription activity of eight *SbGATAs* at the mid-grain filling stage in root, stem, young leaf, mature leaf, flower, fruit, and husk. Values of column chart are expressed as Mean ± SD, the different lowercase letters represent significant differences (*p*<0.05, Duncan test). **(B)** Corresponding correlation hierarchical cluster analysis. Positive number represents positive correlation and negative number indicates negative correlation. The right color scale (−0.60 to 1.00, blue to red) represents the normalized gene expression correlation.

GATAs are a class of transcription factors closely related to growth and development. Therefore, we studied the tissue-specific transcriptional activity of eight *SbGATA* genes coming from different subfamilies in the middle grain-filling stage (**Figure 8A**). We found that the relative expression of all the eight *SbGATAs* was higher in fruit in general, while a few genes also had higher expression in other tissues. For example, the expression level of *SbGATA15* was significantly higher in root, stem and young leaf, and the expression level of *SbGATA04* was higher in mature leaf (**Figure 8A**). Obviously, the expression level of most gene, such as *SbGATA04*, *SbGATA11*, *SbGATA16*, *SbGATA28*, *SbGATA29* and *SbGATA33*, was lower in stem and husk (**Figure 8A**). Further correlation analysis of the eight *SbGATAs* among different tissues (**Figure 8B**) suggested that *SbGATA28* and *SbGATA33* had the highest correlation coefficient with a value of 0.964. Surprisingly, *SbGATA11* showed an extraordinarily significant positive correlation with all genes (p<0.01) except *SbGATA15* (**Figure 8B**). It is worth noting that *SbGATA15* was negatively correlated with five genes (p>0.05) except *SbGATA16* and *SbGATA33* (**Figure 8B**).

### 3.8 Transcriptional activity of *SbGATA* genes in grain-filling stages

**Figure 9:**
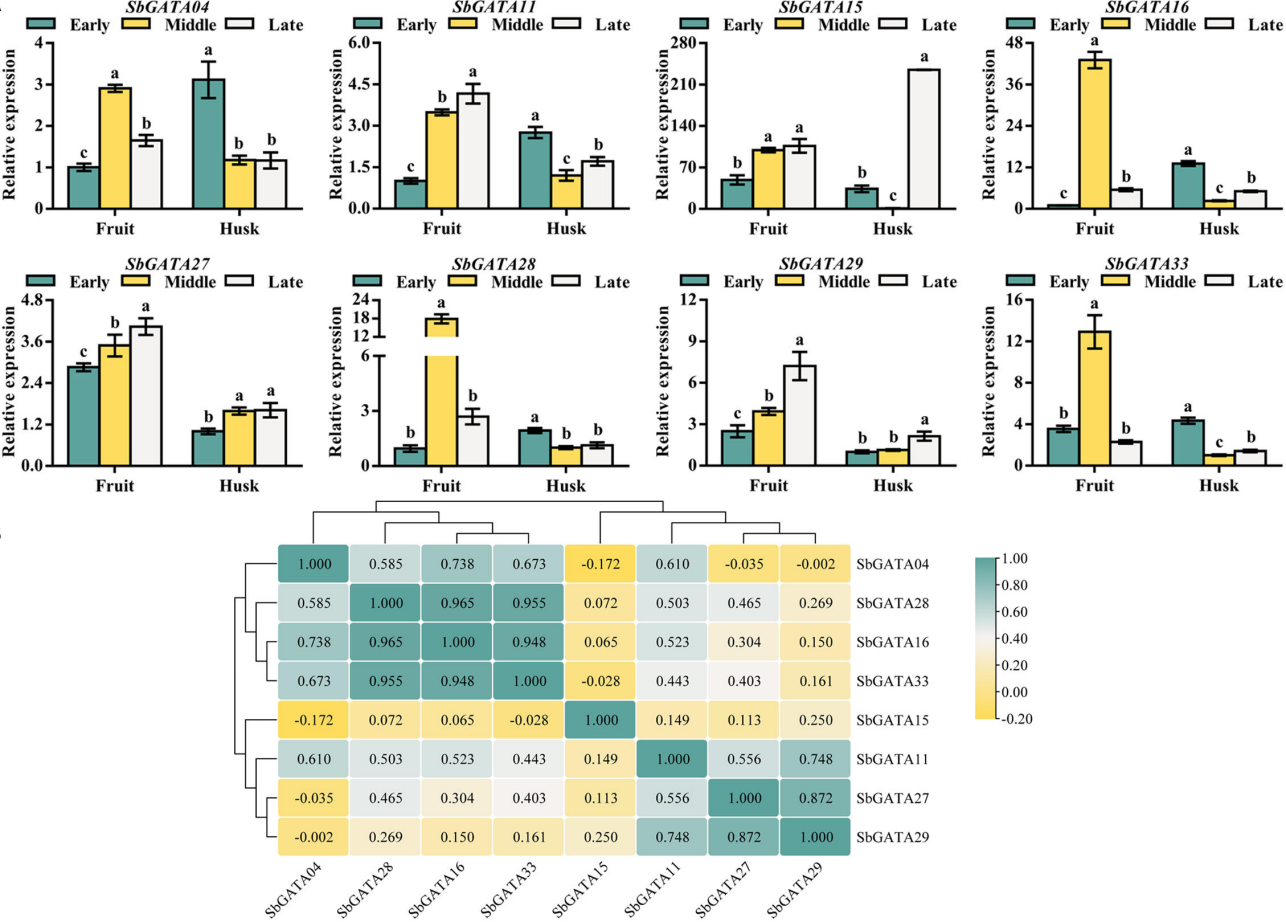
Relative transcriptional activity and corresponding correlation analysis of eight *SbGATAs* (*SbGATA04*, *SbGATA11*, *SbGATA15*, *SbGATA16*, *SbGATA27*, *SbGATA28*, *SbGATA29* and *SbGATA33*) in fruit and husk at different grain-filling stages. **(A)** Relative transcriptional activity of eight *SbGATAs* in the fruit and husk during early, middle, and late grain-filling stages. Values of column chart are expressed as Mean ± SD, the different lowercase letters represent significant differences (*p*<0.05, Duncan test). **(B)** Correlation analysis of relative transcriptional activity between fruit and husk during the grain-filling stage. Positive number represents positive correlation and negative number indicates negative correlation. The right color scale (−0.20 to 1.00, yellow to green) represents the normalized gene expression correlation.

In the previous tissue-specific transcriptional activity study, all the eight tested SbGATAs were found to be highly expressed in fruits, thus the transcriptional activity of *SbGATAs* in the fruit and husk during the early, middle and late grain-filling stage was further investigated (**Figure 9A**). The results showed that *SbGATA04*, *SbGATA16*, *SbGATA28* and *SbGATA33* were highly expressed in fruit in the middle grain-filling stage (**Figure 9A**), while *SbGATA11*, *SbGATA15*, *SbGATA27* and *SbGATA29* were highly expressed in fruit in the late grain-filling stage (**Figure 9A**). Overall, *SbGATAs* were expressed at a higher level in fruits compared to the husk. In the present study, by analyzing the correlation between *SbGATAs* in fruit and husk at different grain-filling stages (**Figure 9B**), we found that most genes were positively correlated with each other (p<0.05), with the highest correlation coefficient between *SbGATA16* and *SbGATA28* (0.964).

### 3.9 Transcriptional activity of *SbGATA* genes in response to abiotic stress treatments

**Figure 10:**
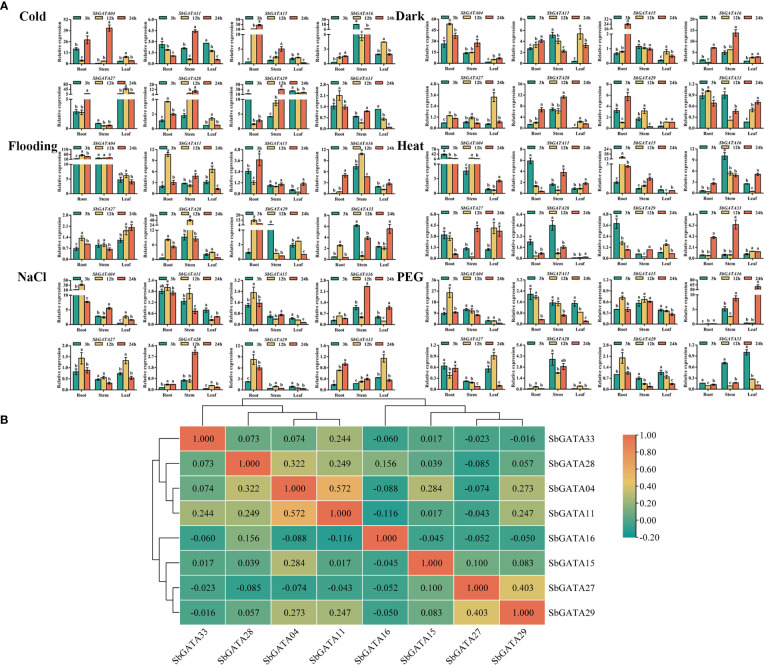
Relative transcriptional activity and corresponding correlation analysis of eight *SbGATAs* (*SbGATA04*, *SbGATA11*, *SbGATA15*, *SbGATA16*, *SbGATA27*, *SbGATA28*, *SbGATA29* and *SbGATA33*) under different stresses (cold, dark, flood, heat, NaCl, and PEG) at the seedling stage. **(A)** Transcriptional activity of eight *SbGATAs* at 3 h, 12 h, and 24 h in root, stem, and leaf. Values of column chart are expressed as Mean ± SD, the different lowercase letters represent significant differences (*p*<0.05, Duncan test). **(B)** Correlation analysis between *SbGATA* expression among the treatments. Positive number represents positive correlation and negative number indicates negative correlation. The right color scale (−0.20 to 1.00, green to orange) represents the normalized gene expression correlation.

Since GATA transcription factors have a vital regulatory role in adversity stress, we initially explored the transcriptional activity of *SbGATAs* under six abiotic stresses, such as cold, dark, flood, heat, NaCl, and PEG (**Figure 10A**) in this study. We found that in general, the relative expression of *SbGATAs* was lower in leaf compared to root and stem, and the relative expression of most *SbGATAs* was mainly concentrated at the initial 12 h after treatment (**Figure 10A**). However, the expression of *SbGATA16* was significantly increased in leaf after 24 h of treatment by PEG (**Figure 10A**). Compared with CK, except dark treatment, *SbGATA16* expression level was down-regulated at 3 h, and except cold treatment, its expression level was down-regulated at 12 h (**Figure 10A**). For *SbGATA28*, the expression in leaf was lower than CK in all treatments, especially heat, NaCl and PEG treatments (**Figure 10A**). In this study, we found that the expression of *SbGATA33* was not higher at 3 h, 12 h and 24 h under most treatments compared to CK, especially the dark, NaCl and PEG treatments (**Figure 10A**). The promoter sequences of *SbGATA04* and *SbGATA27* had low-temperature element and their expression was high under cold treatment (**Figures 4**, **10A**). Meanwhile, *SbGATA04* of subfamily III had generally higher relative expression levels in root under all six abiotic stress treatments, whereas *SbGATA16* of subfamily I had higher relative expression in stem under all treatments (**Figure 10A**). By analyzing the correlation of eight *SbGATAs* under six abiotic stress treatments (**Figure 10B**), we found that the correlation coefficient between all genes was not significant, indicating that the transcriptional activity of any specific *SbGATA* is treatment-dependent and the underlying regulatory mechanisms were different. Surprisingly, *SbGATA16* and *SbGATA27* were negatively correlated with most genes (p>0.05), while *SbGATA04* and *SbGATA11* were significantly positively correlated (p<0.01) and had the largest correlation coefficient with a value of 0.572 (**Figure 10B**).

### 3.10 Transcriptional activity of *SbGATA* genes in response to hormone treatments

**Figure 11:**
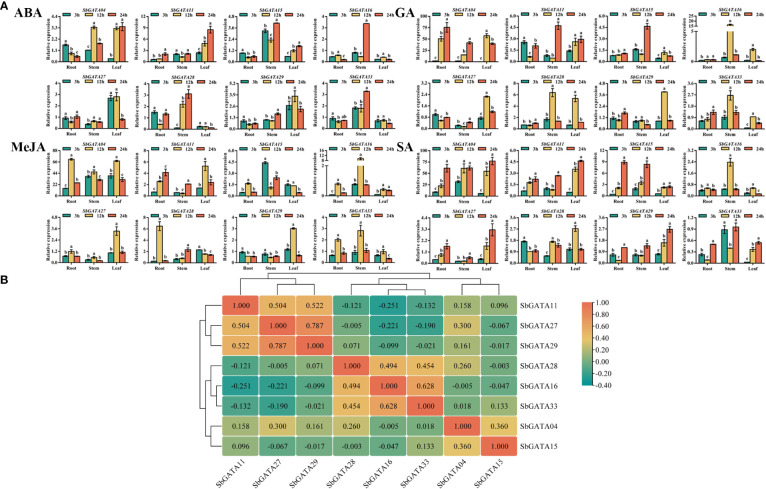
Relative transcriptional activity and corresponding correlation analysis of eight *SbGATAs* (*SbGATA04*, *SbGATA11*, *SbGATA15*, *SbGATA16*, *SbGATA27*, *SbGATA28*, *SbGATA29* and *SbGATA33*) under different hormone treatments (abscisic acid, ABA; gibberellin, GA; methyl jasmonate, MeJA; and salicylic acid, SA) at the seedling stage. **(A)** Relative transcription activity of eight *SbGATAs* in root, stem, and leaf at 3 h, 12 h, and 24 h after hormone treatment. Values of column chart are expressed as Mean ± SD, the different lowercase letters represent significant differences (*p*<0.05, Duncan test). **(B)** Correlation analysis of relative transcriptional activity between different *SbGATAs* after the treatments. Positive number represents positive correlation and negative number indicates negative correlation. The right color scale (−0.40 to 1.00, green to orange) represents the normalized gene expression correlation.

The cis-acting elements in the promoter sequences of *SbGATAs* were analyzed previously and hormone-responsive elements were found to be distributed in most promoters. Therefore, in this paper, the transcriptional response of *SbGATAs* in different subfamilies to hormone treatments were investigated in sorghum seedlings (**Figure 11A**). The results showed that the relative expression levels of *SbGATA04*, *SbGATA11*, *SbGATA16* and *SbGATA28* showed similar trends among the tissues under gibberellin (GA) and salicylic acid (SA) treatments (**Figure 11A**). The *SbGATA16* of subfamily I had similar transcriptional activity under GA, methyl jasmonate (MeJA) and SA treatments, and the *SbGATA11* of subfamily IV had the same transcriptional activity under abscisic acid (ABA), GA and SA treatments (**Figure 11A**). The cis-element of the eight *SbGATA* genes predicted in the previous section had both ABA and MeJA regulated fragments, and all *SbGATA* genes were expressed under GA treatment, with *SbGATA04* highly expressed under MeJA treatment (**Figures 4**, **11A**). The relative expression of *SbGATA04* under the GA, MeJA and SA treatment was the highest overall. The correlation heat map (**Figure 11B**) showed that *SbGATA04* was positively correlated with all genes except *SbGATA16*, and *SbGATA15* and *SbGATA16* were negatively correlated with most genes. The highest correlation coefficient with a value of 0.628 was found between *SbGATA16* and *SbGATA33* (**Figure 11B**).

## 4 Discussion

### 4.1 Identification and characteristics of SbGATA in *Sorghum bicolor*

In this study, a total of 33 *S.bicolor* GATA gene family members (**Table S1**) were identified using various bioinformatics methods and their proteins molecular weight (MW), theoretical isoelectric point (pI) and other physicochemical properties (**Table 1**) were further analyzed. There were significant differences among the 33 SbGATAs in terms of protein primary structure and molecular weight (MW), indicating that *S.bicolor* had different degrees of differentiation in order to adapt to environmental changes during long-term evolution. The pI of most SbGATA proteins (22/33, 66.67%) was between 7 and 9 (**Table 1**), indicating that the SbGATA family tends to be rich in weakly alkaline amino acids, which is consistent with the results of related studies in monocotyledons (Cheng et al., 2021; Lai et al., 2022b) but not in dicotyledons (Yao et al., 2022a), indicating that there are significant differences in gene types between monocotyledonous and dicotyledonous plants. The subcellular localizations of the 33 SbGATAs were predicted using WoLF PSORT, and 26 were located in the nuclear; 5, in the chloroplast; and 2 in the mitochondria (**Table 1**), suggesting that the SbGATAs mainly plays a leading role in controlling transcription within the nuclear.

The 33 SbGATAs were divided into four subfamilies (I, II, III and IV) (**Figure 1A**), which is consistent with most GATA family studies (Du et al., 2022; Feng et al., 2022), indicating that the GATA family is relatively stable during evolution. It is worth noting that SbGATA05 and SbGATA23 formed a single branch in subfamily IV (**Figure 1A**), suggesting that the evolution of *S. bicolor* is more complicated and different from other subfamily members. At the same time, the homology of SbGATA proteins in the same evolutionary branch was high (**Figure 1A**), indicating that they have higher sequence conservation and closer evolutionary relationship. With the exception of a few (SbGATA06 of subfamily IV; SbGATA24, SbGATA25 and SbGATA27 of subfamily I), all SbGATAs contain only one GATA domain, which indicates the 33 SbGATAs are found to be highly conserved (**Figure 1B**). The zinc finger domains of subfamilies I, II and IV all have the CX_2_CX_18_CX_2_C structure, while subfamily III has the CX_2_CX_20_CX_2_C structure (**Figure 1B**), which is consistent with other plant GATA studies (Gupta et al., 2017; Zhang et al., 2018). Interestingly, the SbGATA10 protein of subfamily IV is in short of the CX_2_C structure (**Figure 1B**), suggesting that this protein may perform a new function distinct from other members in subfamily IV, which is not present in some crops (Peng et al., 2021; Yao et al., 2022a). There are different conserved sequences between different subfamilies and some differences in the GATA domain between the same subfamily (**Figure 1B**), allowing the SbGATA proteins to diversify and generate different physiological and biochemical regulatory functions.

Although the gene/protein length, MW, and pI of the *S. bicolor* GATA family are highly variable (**Table 1**), their amino acid motifs and gene structures are relatively conserved (**Figure 2**). The 33 SbGATAs contain 1-7 motifs, and motif 1 is widely distributed in them except SbGATA10, indicating that motif 1 was the Zinc finger GATA motif of this family. The fact that SbGATA10 does not have the motif 1, is consistent with the result that it is lack of CX_2_C conserved structure in the previous multiple sequence alignment (**Figure 1B**). However, different subfamilies had unique conserved motifs and the motifs of SbGATA proteins in the same subfamily are similar (**Figure 2B**), further supporting the functional differences among SbGATA members in different subfamilies, which is consistent with most GATA family studies in other crops (Du et al., 2022; Feng et al., 2022). At the same time, we found that the *SbGATAs* genes of subfamily III and IV are rich in CDS and introns, especially the *SbGATA23* gene of subfamily IV, which contains 8 CDS regions and 7 introns (**Figure 2C**). It has been shown that the higher the number of introns and the longer the gene sequence, the higher the frequency of recombination between genes (Shabalina et al., 2010). The distribution of conserved motifs/gene structures are similar between members of the same subfamily, but differ considerably between subfamilies, suggesting that the classification of each subfamily is accurate, which is consistent with the findings of Manfield et al. (2007) and Yu et al. (2019).

The interaction between SbGATA proteins was also predicted (**Figure 3**) and the result suggested that 22 SbGATA proteins interacted with each other, of which SbGATA33 and SbGATA23 interacted with 12 and 7 SbGATA proteins, respectively, indicating that these two play an important regulatory role in the SbGATA family. Cis-acting elements, including promoters and enhancers, are involved in the regulation of gene expression by binding to trans-acting factors to regulate the activity of target genes (Liu et al., 2019). Plant *GATA* gene is an important gene that regulates light signal transduction by binding to related motifs in the *GATA* promoter sequence (Buzby et al., 1990; Luo et al., 2010). In the present study, the cis-acting elements in the promoter regions of the 33 *SbGATAs* were predicted. The promoter regions of *SbGATAs* genes were found to contain growth and development, physiological regulation, abiotic stress and plant hormone elements (**Figure 4**). Among them, light responsiveness elements (with a count of 377), abscisic acid responsiveness elements (123) and MeJA responsiveness elements (87) were widely distributed (**Figure 4**), supporting that *GATA* genes participate in plant growth and development, stress physiology and hormone signal transduction, which is consistent with Yu et al. (2019) and Peng et al. (2021). Of interest is *SbGATA10*, which has five types of hormone response elements and two types of abiotic response elements (**Figure 4**), suggesting that it is highly sensitive to the environment changes and may participate in multiple physiological and biochemical regulatory mechanisms.

### 4.2 Gene duplication and evolutionary relationship of *SbGATAs* genes in *Sorghum bicolor*

A total of 33 *S. bicolor GATA* genes were identified in this study, while the number of *GATA* genes identified in *A. thaliana*, *G. max* and *O. sativa* are 30, 64 and 35, respectively. This difference may be due to gene recombination, gene duplication, and segment duplication during natural differentiation and evolutionary evolution (Vision et al., 2000; Huang et al., 2021); or due to frequent rearrangements of genes in chromosomal regions, which causes most of the duplicated gene copies are lost or moved to new sites (Zhang et al., 2017), which may also be related to the genome size of each species, such as *S. bicolor* (730 Mb) (Deschamps et al., 2018), *A. thaliana* (125 Mb) (Schneeberger et al., 2011), *G. max* (1.025 Gb) (Shen et al., 2018) and *O. sativa* (466 Mb) (Yu et al., 2005). Genome-wide identification showed that 33 *SbGATAs* were distributed on 10 chromosomes of *S. bicolor*, with each randomly distributed 2-9 *SbGATAs* except Chr 7 (**Figure 5A**), indicating that they have their own distinct role. The presence of tandem duplication genes has, to some extent, provided the basis for the evolution of the GATA transcription factor family (Sykes et al., 1998; Chalhoub et al., 2014). In our study, a tandem duplication gene pair——*SbGATA24* and *SbGATA25*, both of which belong to subfamily I, was identified on Chr 8 (**Figure 5A**; **Table S4**), suggesting that these two may be transcripted together to regulate related biological processes. Meanwhile, nine pairs of *SbGATAs* segment duplication events occurred in the *S. bicolor* GATA family, with five pairs belonging to subfamily II (**Figure 5B**). In addition, synteny analysis of *GATAs* between *S. bicolor* and three monocotyledons (*S. lycopersicum*, *B. distachyon* and *O. sativa*) and three dicotyledons (*A. thaliana*, *V. vinifera* and *G. max*) suggested that the *S. bicolor GATAs* family had the most common genes with *B. distachyon* (39) and *O. sativa* (39) but had less with the dicotyledons (*A. thaliana*, *V. vinifera* and *G. max*) (**Figure 6**; **Table S5**). And the interspecific evolutionary relationships analysis also found that *S. bicolor* GATAs clustered more closely with those of the monocotyledons, such as *B. distachyon* and *O. sativa* (**Figure 7**), which might be not only related to *S. bicolor* among monocotyledonous plants, but also related to the emergence of monocotyledonous and dicotyledonous classifications of angiosperms during long term natural selection and evolution. Among them, *SbGATA07*, *SbGATA12* and *SbGATA28* had synteny homologous genes with all six plants (**Figure 6**; **Table S5**). *SbGATA14* and *SbGATA29* had synteny genes with all three monocotyledons (**Figure 6**, **Table S5**), which indicates a high degree of homology between monocotyledons. And it also shows that the five genes (*SbGATA07*, *SbGATA12*, *SbGATA14*, *SbGATA28* and *SbGATA29*) are present in monocotyledons before differentiation and have important regulatory mechanisms during growth and development. In contrast, *SbGATA02*, *SbGATA05*, *SbGATA08*, *SbGATA10*, *SbGATA16*, *SbGATA20*, *SbGATA25* and *SbGATA26* did not share a common synteny gene with any of the six plants (**Figure 6**; **Table S5**), suggesting that these genes were formed after *Sorghum* differentiation, which has similar results in other crops and other gene family (Sun et al., 2020; Fan et al., 2021b; Yao et al., 2022a). It can be seen that the *S. bicolor GATA* gene family have been amplified to some extent, but that gene loss has also occurred during evolution, suggesting that the lost *GATA* genes may have been replaced by functionally similar genes (Lynch and Conery, 2000), but nevertheless implying that the amplified *S. bicolor GAT*A genes have played an important role in the evolution of *S. bicolor*.

### 4.3 Spatio−temporal expression patterns of the *SbGATA* genes in *Sorghum bicolor*

It was found that *BdGATA13*, which has high homology with the GATA transcription factor GNC, deepened leaf color, delayed flowering period, enhanced drought resistance and promoted primary root development when overexpressed under GA treatment in transgenic arabidopsis (Guo et al., 2021). In rice, the *OsGATA23a* gene is a multi-stress responsive TF with elevated expression levels under salt stress and drought stress (Gupta et al., 2017). These studies show that GATA plays an important regulatory role in plant growth and development, and in response to signal transduction. In the present study, we investigated the spatio-temporal transcription activity of eight *SbGATAs* of different subfamilies in different tissues during fruit development, abiotic stresses and hormone responses. As expected, the transcriptional activity of *SbGATAs* is tissue-dependent, and they are significantly expressed in fruit in general except *SbGATA04* and *SbGATA15*, which had higher expression in young leaf at filling stage. The expression levels of the same genes in different tissues were also different, such as *SbGATA04*, *SbGATA16* and *SbGATA28* (**Figure 8A**). Moreover, the expression of *SbGATAs* in fruit was higher than that in the husk during fruit development, (**Figure 9A**), suggesting that the expression of *SbGATAs* is tissue-specific. The qRT-PCR analysis of *SbGATA* genes in response to abiotic stress treatments revealed that *SbGATA* genes were differentially expressed at different times and under different abiotic stress treatments (**Figure 10A**), with higher expression of *SbGATA04* at the root (**Figure 10A**), indicating that this gene has a strong influence on the regulatory role of the root system in response to the environment and has a strong physiological response to stress. At the same time, it also showed that the members of the *SbGATA* genes family were involved in the stress response process of *S. bicolor*. In the hormone response, some *SbGATAs* showed similar expression trends under ABA, GA or SA treatments. For example, *SbGATA16* showed similar gene expression trends under GA, MeJA and SA treatments (**Figure 11A**). Among them, the relative expression level of *SbGATA04* under GA, MeJA and SA treatments was the highest, generally (**Figure 11A**). The above results showed that the expression patterns of the *SbGATAs* family are diverse in different tissues, at different times and under different environments, indicating that the SbGATA family is functional diversity and plays a key role in tissue development and environmental response.

## 5 Conclusion

In this study, 33 *GATA* genes were systematically identified for the first time from the whole *S. bicolor* genome. These *SbGATA* genes are randomly distributed on 10 chromosomes of *S. bicolor* containing one pair of tandem duplications and nine pairs of segment duplications and are further grouped into four subfamilies (I-IV). The SbGATA proteins have the highest homology with the monocots *B. distachyon* and *O. sativa*. While these SbGATA proteins have markedly different physicochemical properties, a high degree of conservation in protein motif is identified as well as corresponding SbGATA proteins structure. Moreover, these *SbGATAs* have tissue specificity and functional diversity during the growth and development of *S. bicolor*. It not only participates in the transcriptional regulation of *S. bicolor* under abiotic stress, but also is induced by plant hormone signals. Our study provides a foundation and theoretical basis for studying the function and mechanism of the *S. bicolor GATA* gene family during plant growth and development.

## Data availability statement

The entire Sorghum bicolor genome sequence information was from the Phytozome website 513 (https://phytozome-next.jgi.doe.gov/). The Sorghum bicolor materials (Hongyingzi) used in the experiment were supplied by Prof. Cheng Jianping and Ruan Jingjun of Guizhou University. All data analyzed during this study are included in this article and its additional files.

## Author contributions

XY and MZ conceived and designed the research. XY, DL, CM, and WJW performed the experiments. XY, WFW and YF performed the data analysis and wrote the manuscript. JR and JC edited and drafted the manuscript. All authors contributed to the article and approved the submitted version.
